# A review of reported network degree and recruitment characteristics in respondent driven sampling implications for applied researchers and methodologists

**DOI:** 10.1371/journal.pone.0249074

**Published:** 2021-04-15

**Authors:** Lisa Avery, Alison Macpherson, Sarah Flicker, Michael Rotondi

**Affiliations:** Department of Kinesiology and Health Sciences, York University, Toronto, Ontario, Canada; Nanyang Technological University, SINGAPORE

## Abstract

**Objective:**

Respondent driven sampling (RDS) is an important tool for measuring disease prevalence in populations with no sampling frame. We aim to describe key properties of these samples to guide those using this method and to inform methodological research.

**Methods:**

In 2019, authors who published respondent driven sampling studies were contacted with a request to share reported degree and network information. Of 59 author groups identified, 15 (25%) agreed to share data, representing 53 distinct study samples containing 36,547 participants across 12 countries and several target populations including migrants, sex workers and men who have sex with men. Distribution of reported network degree was described for each sample and characteristics of recruitment chains, and their relationship to coupons, were reported.

**Results:**

Reported network degree is severely skewed and is best represented by a log normal distribution. For participants connected to more than 15 other people, reported degree is imprecise and frequently rounded to the nearest five or ten. Our results indicate that many samples contain highly connected individuals, who may be connected to at least 1000 other people.

**Conclusion:**

Because very large reported degrees are common; we caution against treating these reports as outliers. The imprecise and skewed distribution of the reported degree should be incorporated into future RDS methodological studies to better capture real-world performance. Previous results indicating poor performance of regression estimators using RDS weights may be widely generalizable. Fewer recruitment coupons may be associated with longer recruitment chains.

## 1 Introduction

Since its development in 1997, respondent driven sampling (RDS) has become increasingly popular for measuring disease prevalence and correlates of disease in hidden populations [1]. In RDS, social connections among members of these hard to reach target populations are used to propagate recruitment, similar to snowball sampling. However, RDS differs from snowball sampling in two important ways: it requires the collection of additional information, including network size and it creates long (as opposed to wide) recruitment chains. Through the use of coupons with unique codes, the number of people a participant can recruit is restricted. This produces long recruitment chains to ensure that the final sample is independent of the initial recruits. It also allows researchers to trace the recruitment process and collect information on who recruited whom. In addition, as a proxy for their sampling probability, participants are asked about their number of connections in the target population. This additional information enables better estimation of disease prevalence by adjusting for non-random selection probability. Several prevalence estimators which account for the RDS design have been developed; the most commonly reported are the Volz-Heckathorn RDS-II estimator [2] and the Gile successive sampling estimator (SS) [3]. Gile et al. [4] have recently reviewed the statistical advances in RDS and give a thorough overview of the available estimators. A number of studies have evaluated the performance of estimators and found that none are uniformly superior [5–10]. The accuracy of the variance estimates is still unclear [2, 7, 11], and depends on network and sampling conditions.

Reported network degree (hence force referred to simply as degree) is an important variable in RDS studies. Degree refers to the number of ties an individual has to others in the target population. Individuals with more network connections are more likely to be recruited into an RDS study, so participants’ reported degree is a proxy measure of sampling probability. Details on the distribution of degrees in RDS simulation studies is scarce, but has been recently modelled as a Poisson process [10], or with the more flexible Conway-Maxwell-Poisson distribution [12]. Empirical research presented by Killworth et al. [13] suggests that social networks have a right-skewed distribution and their histograms of network degree suggest a log-normal distribution. Our recent finding [14] that weighted regression methods performed poorly when the reported network degree was highly skewed raised the question of whether those data were unique or if highly skewed degree distributions are common. Preliminary analyses suggested that, if degree is normally distributed, weighted regression may perform much better. Therefore, the question of how degrees are distributed is of great practical importance: if skewed distributions are common then our recommendation for regression analyses remains not to weight observations, otherwise, more work is necessary to determine appropriate regression strategies. Our previous regression work was motivated by an RDS sample of Indigenous people living in Toronto, Canada. These participants reported degrees that were extremely skewed and appeared log normally distributed. This distribution resulted in participants with low reported degree being assigned very high weights. These acted as leverage points in the regression analysis, and resulted in poor regression parameter coverage rates [14].

To continue to make improvements to the quality of inferences for RDS samples, it is necessary to understand the real-world samples to which these methods are applied. Much work has been dedicated to evaluating RDS estimates by simulation, which requires some assumption regarding degree distribution. The objectives of this study were two-fold: 1) to describe the distribution of reported degree distributions in real-world samples from a variety of geographies and 2) to better inform RDS methodology researchers on how to model degree distributions for methodological studies.

## 2 Methods

### 2.1 Search strategy

Authors of recently published papers were contacted and asked to share study data on reported degree and recruitment chains. The PubMed database was searched for papers using RDS published in English, between 1 January 2019 and 31 August 2019 using the following search term: *((“respondent driven sampling”[Title/Abstract]) AND (“2019/01/01”[Date—Publication]*: *“2019/08/31”[Date—Publication])) AND “english”[Language]*. One hundred six results were returned; there were three additional manuscripts in the author’s reference database published in this period, so 109 manuscripts were examined for eligibility. There was one duplicate manuscript, two protocol studies, three studies employing non-traditional RDS techniques without degree estimates, a methods based manuscript with no sample and one study with a sample size too small (n = 36) to examine degree distribution. From the remaining 101 manuscripts 59 unique author groups were identified and contacted, 15 (25%) agreed to share data on 53 distinct RDS samples. Research ethics approval was not obtained because all data were anonymised prior to being shared. Data on the chain linkages and personal network sizes were sought, no demographic data was collected. Details of the data available from these studies are presented in Table 1.

**Table 1:** Description of studies contributing information about reported degree and recruitment chains.

Article & Sample ID(s)	Target Population Description	Study Setting	Period	Sample Size	Degree Question*	Funding Information
Burton 2019 (s53)	African, Caribbean and Black Youth	Windsor, Canada	2012–2015	511	In a typical week, how many African, Caribbean or Black youth (aged 16–25 years), living in Windsor or Essex County, do you interact with? This could be in person, by phone, or using the internet.	Canadian Institutes of Health Research Operating Grant—Community Based Research HIV/AIDS
Cucciare 2019 (s22)	Rural stimulant users	Arkansas, Kentucky and Ohio, United States	2002–2008	aggregate data across areas analysed (n = 243)	How many other drug users do you know in your community	US National Institute on Drug Abuse (R01 DA15363, R01 DA14340)
Dickson-Gomez 2019 (s18)	Crack users	San Salvador, El Savador	2011–2016	2017 (summary data provided, raw data unavailable)	Number of crack users seen in the past 30 days	US National Institute on Drug Abuse (R01DA020350) and the National Institute on Mental Health (5P30MH57226)
Kitching 2019 (s52)	Indigenous people	Toronto, Canada	2015–2016	917	Approximately how many Aboriginal people do you know (ie, by name and that know you by name) who currently live, work or use health and social services in Toronto?	Canadian Institutes of Health Research Operating Grant
Lachowsky 2019 (s1-s3)	Men who have sex with men	Montreal, Toronto and Vancouver, Canada	2011–2016	Distinct samples for Montreal (n = 1179), Toronto (n = 517) and Vancouver (n = 753)	How many men who have sex with men aged 16 years or older, including trans men, do you know who live or work in the [Metro Vancouver/Greater Toronto/Metro Montreal] area (whether they identify as gay or otherwise)? This includes gay/bi guys you see or speak to regularly.	National Institute on Drug Abuse (R01DA031055–01A1) and the Canadian Institutes for Health Research (MOP-107544, 143342, PJT-153139)
Meyer 2019 (s45-s47)	Migrant workers	Mae Sot, Thailand	2011–2012	Three distinct groups of migrant workers: argicultural (n = 203), factory (n = 258) and sex (n = 128)	How many migrant (agricultural/factory/sex) workers who are over 18 and currently working in your job from Burma dow you known and speak to in the past week?*	Office to Monitor and Combat Trafficking in Persons (S-SGTIP-11-GR-0024)
Morozova 2019 (s37-s38)	Injection drug users	Mykolaiv and Odesa, Ukraine	2011–2013	Aggregate data across cities was supplied for surveys in 2011 (n = 9050) and 2013 (n = 9486). Data on recruitment chains not available	How many people do you know (by name, and they know you by name) who injected drugs during the last 30 days, and you have seen in the past 30 days?	US National Institute on Drug Abuse (grant R01 DA026739), and the Global Fund to Fight AIDS, Tuberculosis and Malaria
Okiria 2019 (s42-s43)	Female sex workers	Nimule and Juba, South Sudan	2016–2018	Distinct samples for Nimule (n = 407) and Juba (n = 841)	How many people do you know (by name, and they know you by name) who injected drugs during the last 30 days, and you have seen in the past 14 days?*	US President’s Emergency Plan for AIDS Relief through the Centers for Disease Control and Prevention (1U2GGH000678)
Otiashvili 2019 (s19)	Injection drug users	Tbilisi, Georgia	2018	149	How many people who have lived in Tbilisi for at least a year do you know that use drugs, who you can seen personally in the past month and are not in the needle and syringe service that you think you could recruit into the study?*	French Ministry of Europe and Foreign Affairs (Grant No. 17SANIN207)
Raymond 2019 (s48-s50)	Transgender women	San Francisco, United States	2010–2016	Three distinct surveys from 2010 (n = 314), 2013 (n = 233) and 2016 (n = 312)	How many other transwomen do you know and have seen in the past one month that you would be willing to give a coupon to?*	US National Institures of Health (1R01 MH109397)
Samkange-Zeeb 2019 (s51)	General Population	Bremen, Germany	2017	115	How many adults who live in your neighbourhood do you know who you have seen in the last four weeks?	Leibniz Institute for Prevention Research and Epidemiology
Solomon 2019 (s4-s15, s20-s35)	Men who have sex with men and injection drug users	Cities across India	2012–2013	Data were available separately for the 22 sites and ranged from 459–1002 for a total of 11,995 MSM and 13,942 PWID	How many (MSM/PWID) have you seen at least once in the past 30 days?	US National Institutes of Health and the Elton John AIDS Foundation
Stoicescu 2018 (s36)	Women Who Inject Drugs	Greater Jakarta	2014–2015	731	How many female friends or acquaintances do you know (you know their name and they know yours), who have injected drugs in the past year, are 18 years or older, and reside in Greater Jakarta or Bandung, and who you would be able to contact right now?	Canadian Institutes of Health Research (Grant No. 314721), Pierre Elliott Trudeau Foundation, Asian Network of People Living with HIV and Australian Injecting and Illicit Drug Users League
Weikum 2019 (s16-s17, s39-s41)	Men who have sex with men/ transgender women and female sex workers	Hagen, Lae and Port Moresby, Papua New Guinea	2015–2016	Data were available separately for three cities who recruited FSW (Hagen, n = 709, Laen = 709 and Port Moresby n = 670) and two cities who recruited MSM/TGW (Hagen n = 111 and Port Moresby n = 400)	How many women do you know who have sold or exchanged sex for money or goods in the last six months, who live in Hagen aged 12 or older who you’ve seen in the past two weeks?*	Government of Australia, the Global Fund to Fight AIDS, TB and Malaria, and the President’s Emergency Plan for AIDS Relief (PEPFAR) through the Centers for Disease Control and Prevention (CDC) under the terms of Cooperative Agreement Number 1 U2G GH001531–01
Weinmann 2019 (s44)	Syrian immigrants	Munich, Germany	2017	195	How many Syrians living in Munich or Upper Bavaria do you know?	Center for International Health (CIH) at LMU Munich

* Questions paraphrased by omitting the nesting structure, as in Table 2.

### 2.2 Analysis

For each sample, a number of distributions were investigated: the Poisson, geometric, negative binomial, normal and log normal distributions using the *fitdistrplus* package in R [15] and the discrete q-exponential, Poisson-lognormal, Conway-Maxwell-Poisson (CMP), Yule and Waring distributions using the *degreenet* package [16]. Fellows [10] used a Poisson distribution to simulate degree distribution; the Poisson-lognormal, CMP, geometric and negative binomial, discrete q-exponential, Yule and Waring are all more general discrete distributions that allow for extra dispersion. McCreesh et al. [9] reported a network distribution that was ‘approximately normal with a slight positive skew’, and raw plots suggested a skewed distribution so the continuous normal distribution was fit to both the raw and log-transformed reported degree. Fit was assessed using the BIC criterion, with smaller values indicating better fit, and by visual inspection of the raw data and fitted curves. Data from studies collected across multiple sites or years were left disaggregated. Participants whose reported network degree was missing were removed from the analysis. Those who reported a network degree of zero were recoded to 1, since in order to be recruited into the study, they needed to know at least one other member of the population. For each sample and participant, the wave that the participant was recruited into, and the identifier of the seed the participant was recruited from were determined. This data was used to examine the distribution of waves across studies and to determine if the reported degree of the seeds was correlated with the total number of participants in the seeds clusters. To give an indication of how effective most RDS studies may be in achieving samples independent of the initial seeds, recruits were ordered by wave and the wave of the median recruit was determined for each sample. This indicates the minimum distance from seeds for at least 50% of the sample. The ease with which RDS chains propagated was investigated by calculating the number of waves recruited for each seed and the number of recruits for every participant, across all studies.

To determine how frequently the population of available recruits is substantially depleted by the sampling process we used a method similar to that reported by Gile et al. [17] and Crawford et al. [18]. These authors regressed 1:n (with n representing the sample size) against the time-ordered reports of network degree to determine if reported degree decreases monotonically with time. Such a decline would be expected if the available recruits were indeed being depleted by the sampling process. Our approach was similar: the wave number (as a proxy for recruitment order) was regressed on the natural logarithm of the reported degree.

Participating studies were asked to report the question(s) that were used to elicit the number of ties to others in the population (their reported degree). Samples were classified into two groups: those that used a single question to define ties and those that used at least two nested questions (see Table 2 for examples). Because the data are observational, formal statistical tests were not applied, but the reported degree was plotted for different populations for studies with single vs. nested questions to explore the effect of tie definition.

**Table 2:** Examples of different strategies for defining ties among population members used to elicit reported network degree from respondents.

Defining ties using a single question	Defining ties using nested questions
How many other drug users do you know in your community?	How many migrant sex workers who are over 18 and are currently or recently working in your job from (*county*) do you know? Of these people from above, how many know you? Of these people who know you, how many did you see in the past week? Of those people you saw, how many did you speak to in the past week?

## 3 Results

Data from 15 groups, containing 53 distinct RDS samples from North and Central America, Europe, Africa and Asia were collected. These samples mainly targeted four types of populations: men who have sex with men, drug users, female sex workers and migrants. In addition, there were samples of transgender women, Indigenous people, youth of colour and one general population sample. The shape of the reported degree distributions was remarkably similar across population type and geography. Table 1 details the location and timing of the studies as well as the questions asked to elicit reported degree.

### 3.1 Distribution of reported degree

**Fig 1:**
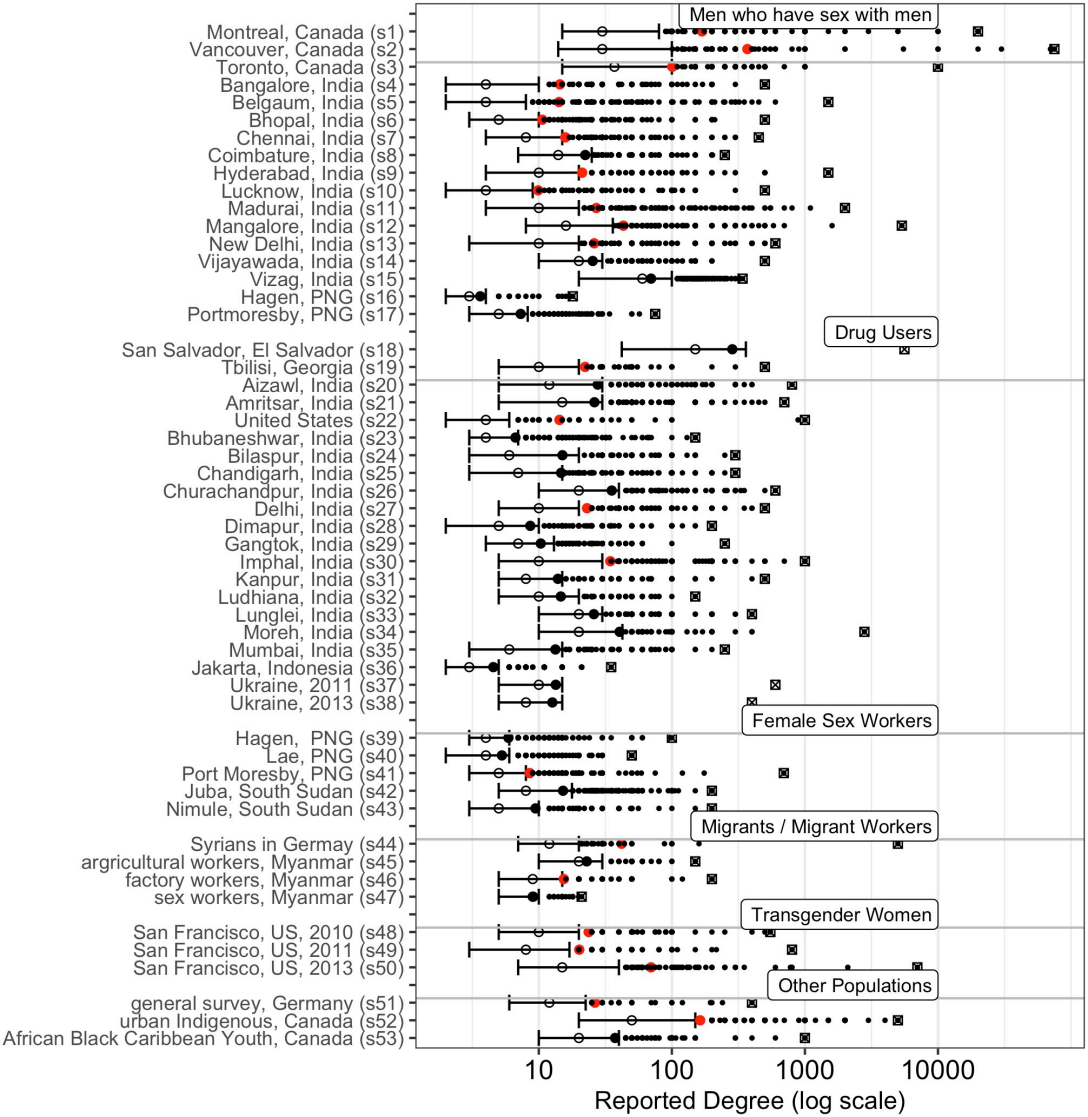
Reported degree distributions across samples. Distribution of reported degree across all samples from various target populations. Bars delineate the interquartile range, the mean is represented by a filled red circle, the median by an open circle and the maximum reported degree by a square box. Small dots indicate all reports above the interquartile range.

**Fig 2:**
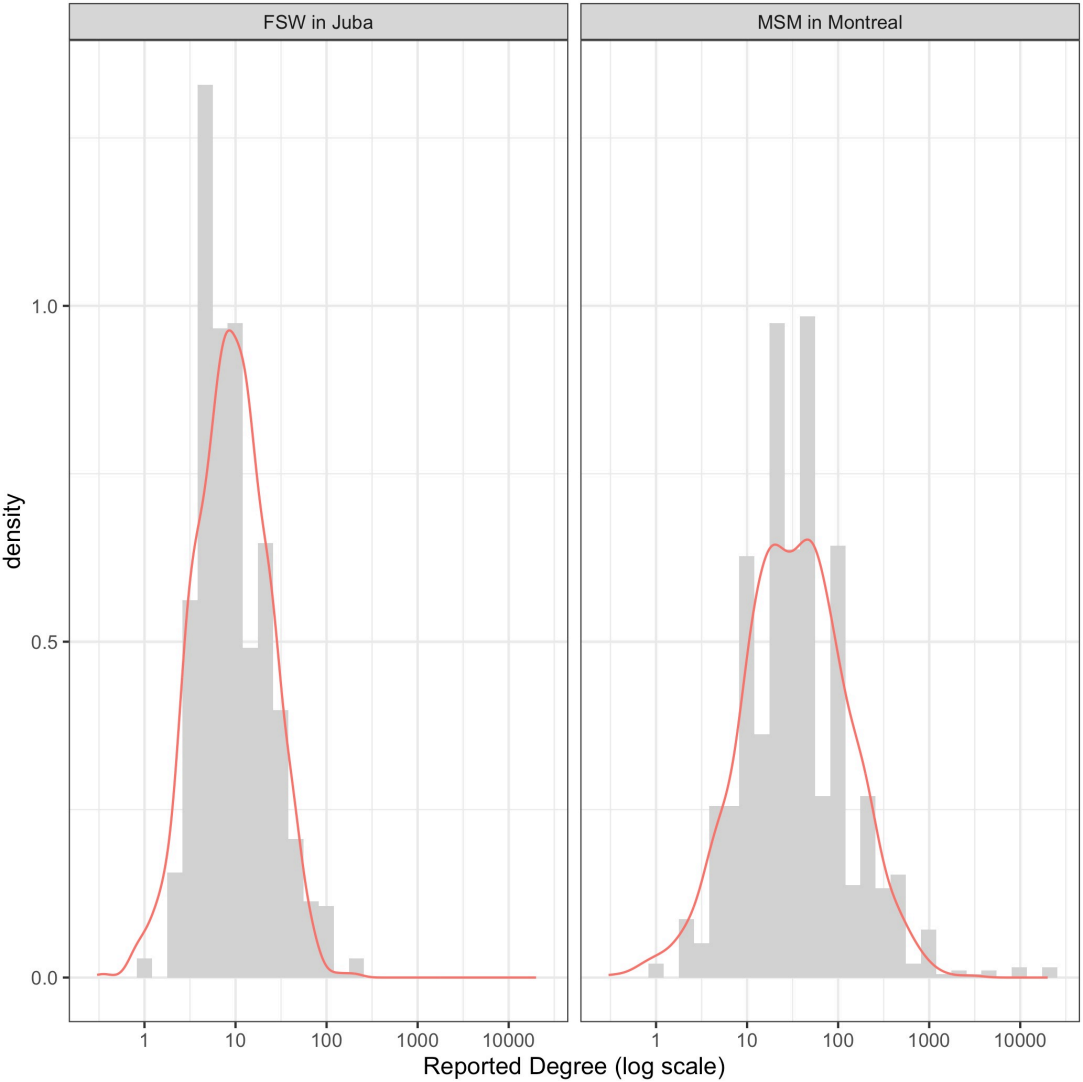
Example distribution densities. Distribution of reported network degree for MSM from Montreal, Canada (n = 1179) and FSW from Juba, South Sudan (n = 846). Both samples are best described by the normal distribution on log-transformed degree.

Under a criteria of minimising the Bayesian information criteria (BIC), the log-normal distribution was the best fit to the data, for all samples. The Conway-Maxwell-Poisson and Poisson models were consistently a poor fit for the network degree data. Fig 1 illustrates the extreme skewness of the reported degrees; the mean (filled circle), median (open circle), interquartile range and maximum reported degree are shown. The mean is always greater than the median and is frequently greater than the 75th percentile. Maximum reported degree is often an order of magnitude larger than the median degree. Very large reported degrees (>1000) are not uncommon, particularly among MSM. Fig 2 illustrates the reported degree (on log scale) and the expected density under a normal distribution on the log of the reported degree for two large samples: MSM in Montreal, Canada (n = 1179) and FSW in Juba, South Sudan (n = 846). Table 3 describes the distribution of the raw and log-transformed degrees and Table 4 indicates the rank of the fit of each candidate degree within each sample, ordered by the BIC statistic.

**Table 3:** Distribution of raw reported degree and log-transformed degree across samples.

Sample		Raw Degree				Log-transformed degree		
Men who have sex with men	N	mean	median	sd	IQR	mean	median	sd
Montreal, Canada (s1)	1179	168.7	30	1153.0	15–80	3.6	3.4	1.3
Vancouver, Canada (s2)	753	369.8	30	3990.6	14–100	3.6	3.4	1.4
Toronto, Canada (s3)	517	100.9	37	454.7	15–100	3.6	3.6	1.3
Bangalore, India (s4)	997	14.4	4	40.8	2–10	1.7	1.4	1.2
Belgaum, India (s5)	998	14.2	4	62.5	2–8	1.4	1.4	1.3
Bhopal, India (s6)	1000	10.6	5	24.3	3–10	1.7	1.6	1.0
Chennai, India (s7)	1002	15.8	8	30.6	4–15	2.1	2.1	1.1
Coimbature, India (s8)	1001	22.3	14	28.6	7–25	2.6	2.6	1.0
Hyderabad, India (s9)	998	21.2	10	61.6	4–20	2.2	2.3	1.2
Lucknow, India (s10)	1000	9.9	4	25.0	2–9	1.5	1.4	1.1
Madurai, India (s11)	996	27.1	10	93.3	4–20	2.3	2.3	1.3
Mangalore, India (s12)	1002	43.2	16	186.4	8–36	2.8	2.8	1.3
New Delhi, India (s13)	997	26.1	10	59.3	3–20	2.2	2.3	1.4
Vijayawada, India (s14)	1002	25.4	20	28.9	10–30	2.9	3.0	0.9
Vizag, India (s15)	1002	69.8	60	58.9	20–100	3.8	4.1	1.1
Hagen, PG (s16)	111	3.6	3	3.3	2–4	1.0	1.1	0.7
Portmoresby, PG (s17)	400	7.3	5	8.1	3–8	1.6	1.6	0.9
Drug Users								
San Salvador, El Salvador (s18)	2107	284.5	150		42–360			
Tbilisi, Georgia (s19)	149	22.3	10	51.6	5–20	2.3	2.3	1.2
Aizawl, India (s20)	997	27.7	12	47.9	5–30	2.4	2.5	1.4
Amritsar, India (s21)	929	26.2	15	47.4	5–30	2.5	2.7	1.3
United States (s22)	243	14.3	4	85.8	2–6	1.3	1.4	1.1
Bhubaneshwar, India (s23)	925	6.7	4	11.2	3–7	1.5	1.4	0.8
Bilaspur, India (s24)	982	15.1	6	25.2	3–20	2.0	1.8	1.2
Chandigarh, India (s25)	930	14.7	7	25.9	3–15	2.0	1.9	1.2
Churachandpur, India (s26)	1000	35.3	20	51.1	10–40	2.9	3.0	1.1
Delhi, India (s27)	990	23.1	10	42.0	5–20	2.5	2.3	1.1
Dimapur, India (s28)	997	8.6	5	15.0	2–10	1.5	1.6	1.1
Gangtok, India (s29)	1002	10.3	7	12.1	4–13	2.0	1.9	0.8
Imphal, India (s30)	998	34.5	10	73.2	5–30	2.6	2.3	1.3
Kanpur, India (s31)	968	13.9	8	30.8	5–15	2.1	2.1	0.9
Ludhiana, India (s32)	866	14.6	10	14.0	5–20	2.3	2.3	1.0
Lunglei, India (s33)	997	25.9	20	32.0	10–30	2.8	3.0	1.0
Moreh, India (s34)	459	40.5	20	136.4	10–43	3.1	3.0	1.0
Mumbai, India (s35)	902	13.3	6	20.5	3–15	1.9	1.8	1.2
Jakarta, Indonesia (s36)	731	4.6	3	3.9	2–5	1.2	1.1	0.8
Ukraine, 2011 (s37)	9050	13.4	10	18.5	5–15			
Ukraine, 2013 (s38)	9486	12.6	8	17.4	5–15			
Female Sex Workers								
Hagen, PG (s39)	709	5.9	4	7.6	3–6	1.5	1.4	0.7
Lae, PG (s40)	709	5.3	4	5.6	2–6	1.3	1.4	0.8
Port Moresby, PG (s41)	670	8.6	5	28.6	3–8	1.7	1.6	0.8
Juba, South Sudan (s42)	846	15.2	8	21.7	5–18	2.2	2.1	0.9
Nimule, South Sudan (s43)	407	9.4	5	15.6	3–10	1.8	1.6	0.8
Migrants, Migrant Workers								
Syrians in Germay (s44)	195	42.0	12	357.4	7–20	2.5	2.5	0.9
argricultural workers, Myanmar (s45)	258	22.9	20	20.9	10–30	2.8	3.0	0.9
factory workers, Myanmar (s46)	203	15.4	9	24.5	5–15	2.2	2.2	0.9
sex workers, Myanmar (s47)	128	9.1	9	4.3	5–10	2.1	2.2	0.5
Transgender Women								
San Francisco, US, 2010 (s48)	314	23.7	10	53.6	5–20	2.3	2.3	1.2
San Francisco, US, 2011 (s49)	233	20.1	8	58.7	3–17	2.1	2.1	1.2
San Francisco, US, 2013 (s50)	312	69.7	15	419.3	7–40	2.8	2.7	1.4
Other Populations								
general survey, Germany (s51)	115	26.4	12	50.9	6–23	2.6	2.5	1.1
urban Indigenous, Canada	917	163.2	50	391.3	20–150	4.0	3.9	1.4
African Black Caribbean Youth, Canada	511	37.5	20	75.0	10–40	3.1	3.0	1.0

**Table 4:** Rank of the fit of each distribution to the sample data, scored by the Bayesian information criterion.

Population	Normal on Log-Transformed Degree	Discrete Q Exponential	Waring	Geometric	Yule	Negative Binomial	Conway-Maxwell Poisson	Normal	Poisson	Poisson-Lognormal
**Men who have sex with men**										
Montreal, Canada	1	2	3	5	4	6	8	7	9	10
Toronto, Canada	1	2	3	4	5	6	8	7	9	10
Vancouver, Canada	1	3	2	5	4	6	8	7	9	10
Bangalore, India	1	2	3	5	4	6	8	7	9	10
Belgaum, India	1	2	3	5	4	6	8	7	9	10
Bhopal, India	1	2	3	5	4	6	8	7	9	10
Chennai, India	1	2	3	4	5	6	8	7	9	10
Coimbature, India	1	2	3	4	6	5	8	7	9	10
Hyderabad, India	1	2	3	5	4	6	8	7	9	10
Lucknow, India	1	2	3	5	4	6	8	7	9	10
Madurai, India	1	2	3	5	4	6	8	7	9	10
Mangalore, India	1	2	3	4	5	6	8	7	9	10
New Delhi, India	1	3	2	5	4	6	8	7	9	10
Vijayawada, India	1	2	3	4	7	5	8	6	9	10
Vizag, India	1	2	5	4	7	3	8	6	9	10
Hagen, PNG	1	2	3	6	4	5		8	7	9
Portmoresby, PNG	1	3	4	5	7	6	2	8	9	10
Drug Users										
United States	1	2	3	5	4	6	8	7	9	10
Aizawl, India	1	3	2	4	6	5	8	7	9	10
Amritsar, India	1	2	3	4	5	6	8	7	9	10
Bhubaneshwar, India	1	3	4	6	5	7	2	8	9	10
Bilaspur, India	1	3	2	5	6	7	4	8	9	10
Chandigarh, India	1	2	3	4	5	6		7	8	9
Churachandpur, India	1	2	3	4	6	5	8	7	9	10
Delhi, India	1	2	3	4	5	6	8	7	9	10
Dimapur, India	1	2	3	5	4	6		7	8	9
Gangtok, India	1	3	4	5	7	6	2	8	9	10
Imphal, India	1	2	3	4	5	6	8	7	9	10
Kanpur, India	1	2	3	4	5	6	8	7	9	10
Ludhiana, India	1	2	3	5	7	4		6	8	9
Lunglei, India	1	2	3	4	6	5	9	7	8	10
Moreh, India	1	2	3	4	5	6	8	7	9	10
Mumbai, India	1	3	2	4	5	6		7	8	9
Tbilisi, Georgia	1	2	3	4	5	6	8	7	9	10
Jakarta, Indonesia	1	3	4	7	6	5	2	8	9	10
Female Sex Workers										
Hagen, PNG	1	3	4	6	5	7	2	8	9	10
Juba, South Sudan	1	3	4	5	6	7	2	8	9	10
Lae, PNG	1	3	4	7	5	6	2	8	9	10
Nimule, South Sudan	1	2	3	5	4	6		7	8	9
Port Moresby, PNG	1	2	3	5	4	6	8	7	9	10
Migrants / Migrant Workers										
factory workers, Myanmar	1	2	3	4	5	6		7	8	9
argricultural workers, Myanmar	1	3	5	4	7	2	8	6	9	10
sex workers, Myanmar	1	6	7	8	9	2	3	4	5	10
Syrians in Germay	1	2	3	5	4	6		7	8	9
Transgender Women										
San Francisco, US, 2010	1	2	3	5	4	6	8	7	9	10
San Francisco, US, 2011	1	2	3	5	4	6	8	7	9	10
San Francisco, US, 2013	1	2	3	5	4	6	8	7	9	10
Other Populations										
African Black Caribbean Youth, Canada	1	2	3	4	5	6	8	7	9	10
General Survey, Germany	1	2	3	4	5	6	9	7	8	10
Urban Indigenous, Canada	1	2	3	4	5	6	8	7	9	10

**Fig 3:**
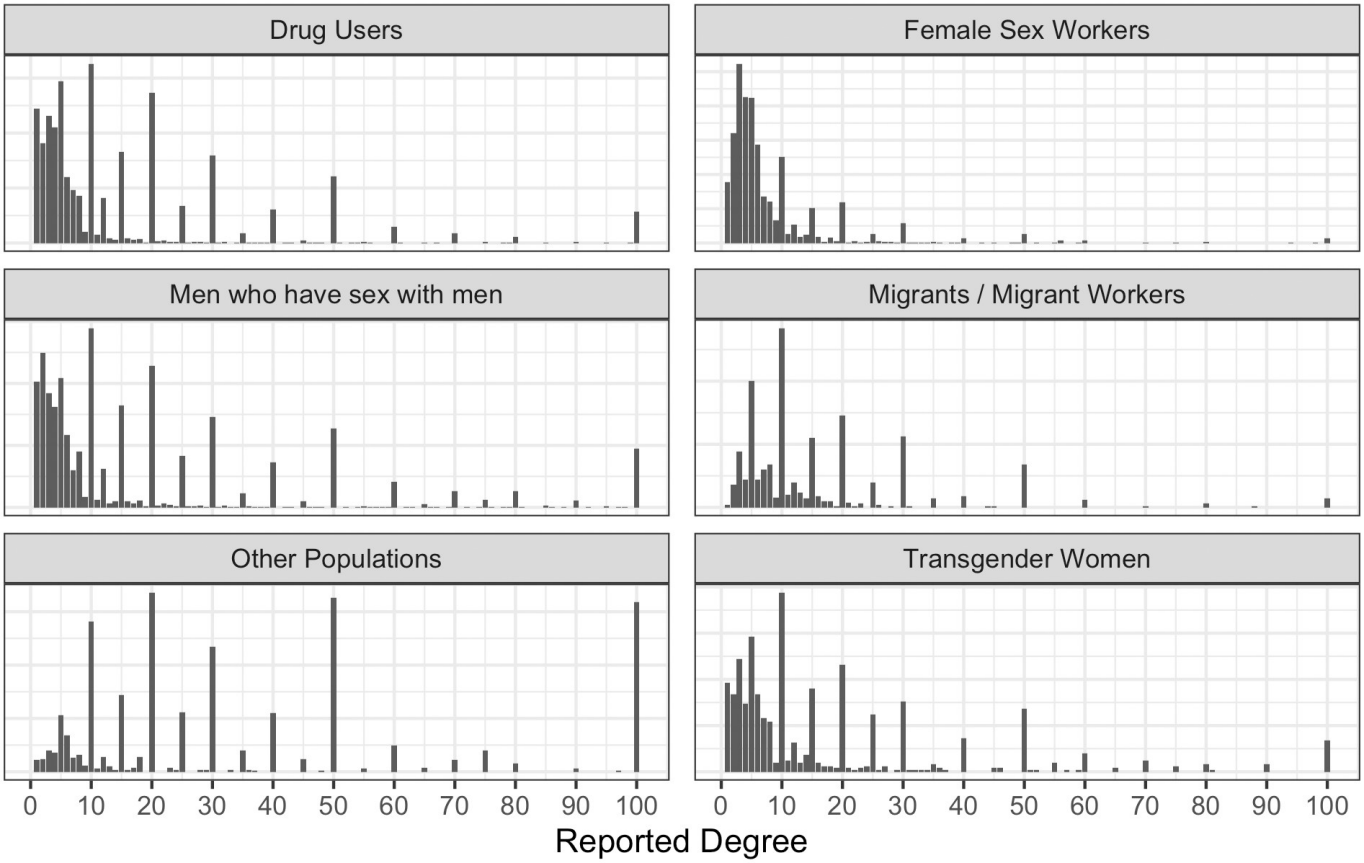
Distribution of degree reports < 100. Relative frequency of reported degree for various populations, aggregated across samples. Only reported degrees up to 100 are shown.

Fig 3 shows the relative frequency of reported degrees across various population types, aggregated across samples, for degrees up to 100. Reported degree, when greater than fifteen, is commonly reported in multiples of five or ten. Of the 12,492 reported degrees greater than fifteen, 81.8% were rounded to the nearest ten, 11.6% were rounded to the nearest five and only 6.6% ended in neither a zero nor a five. This rounding is evident in Fig 3, the general shape and spread of the observed degrees follow a log normal distribution, but degrees ending in 0 are reported much more frequently than expected.

### 3.2 Recruitment characteristics

**Fig 4:**
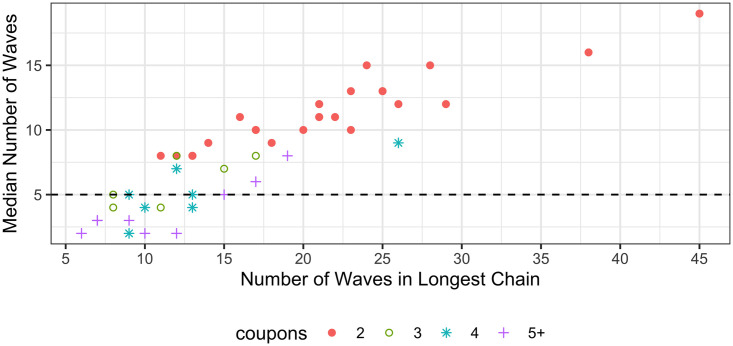
Recruitment waves. Relationship between the number of waves in the longest recruitment chain and the median number of waves across all studies. Each study sample is represented by one data point.

**Fig 5:**
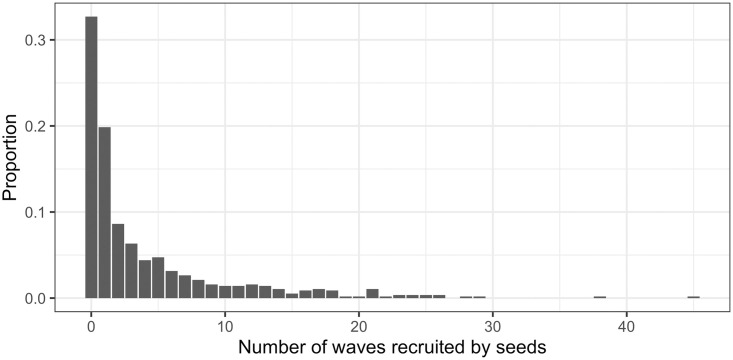
Recruitment waves by seed. Number of waves recruited by seeds (n = 549) across all studies.

**Fig 6:**
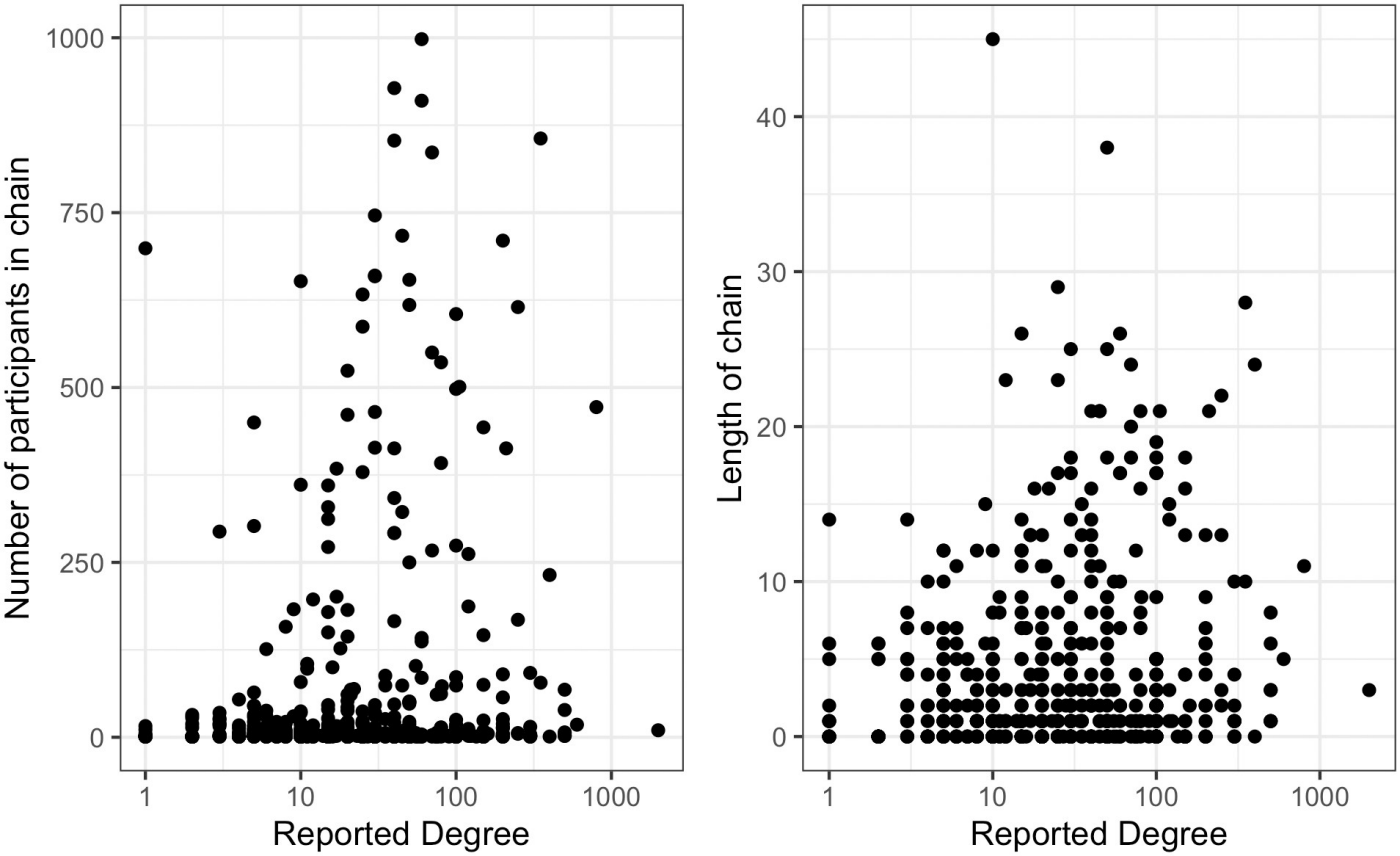
Seed degree and recruitment. Relationship between reported degree of seed and the length and number of participants in recruitment chains.

The number of waves recruited by each seed, corresponding to the length of recruitment chains was calculated, across all samples. Approximately one-third of seeds were unsuccessful in recruiting participants into the study, one-third of seeds produced recruitment chains of between one and three waves and the final third produced chains four waves or longer. Fig 4 examines the relationship between the number of waves in the longest recruitment chain and the wave of the median recruit. Fig 5 illustrates the distribution of recruitment chain length for all seeds, over 30% of seeds did not recruit. Fig 6 plots seed degree against both chain length and number recruited and indicates seed degree is not correlated with recruitment success. Recruitment per person (including seeds) was summarised across all studies; of the 36,547 participants, 47% did not recruit, 15% recruited one person, 34% recruited two people and 4% recruited three or more.

### 3.3 Reported degree and tie definition

**Fig 7:**
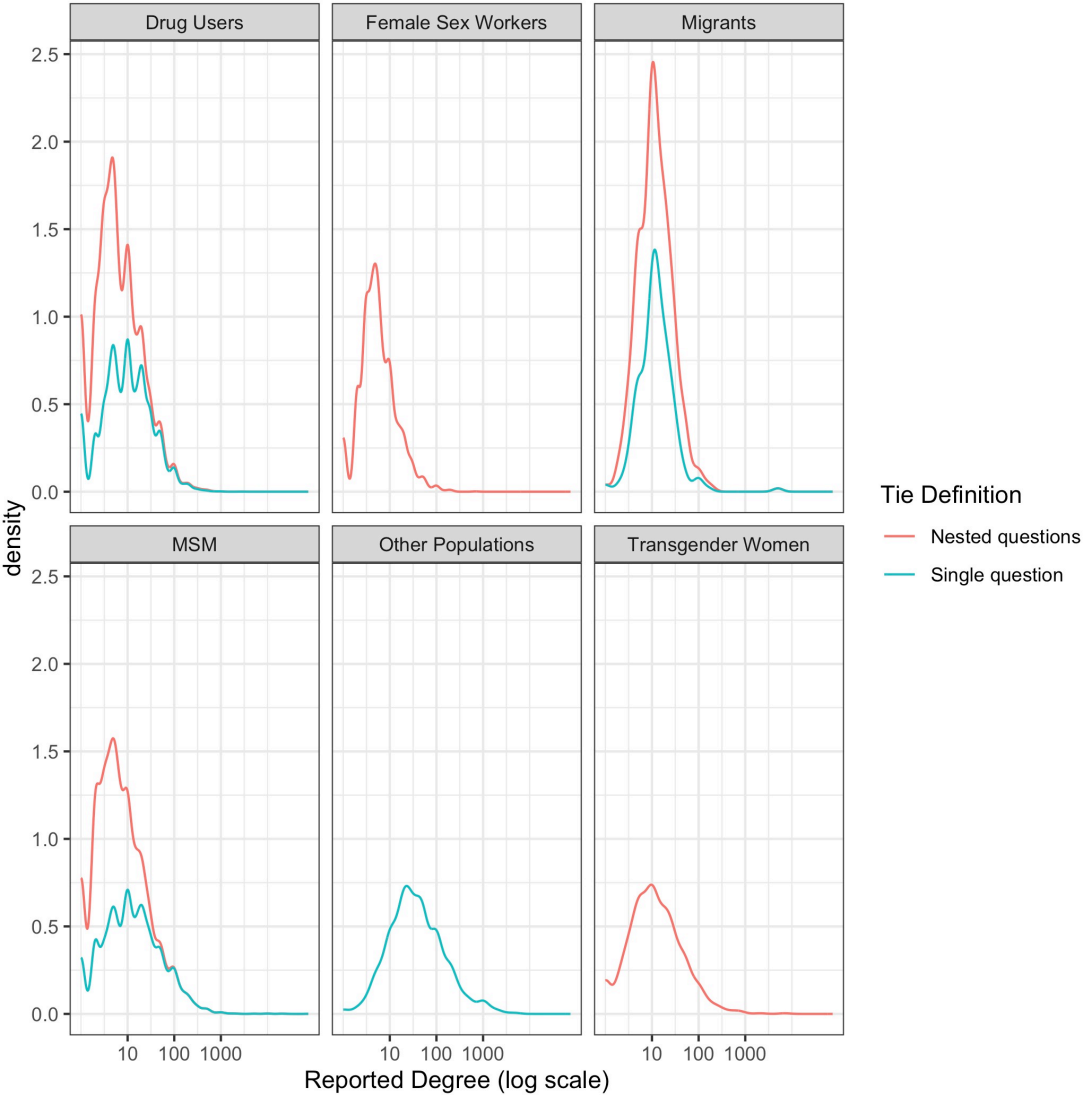
Network degree by tie definition. Distribution of reported network degree based on population type and tie definition.

Samples were classified into two groups based on how network ties were defined: those that used a single question and those that used two or more nested questions (as in Table 2). Fig 7 illustrates the reported degree, across population types, by tie definition. Because of the variability in tie definition across groups, no formal statistical analyses were conducted.

### 3.4 Trend in degree over time

**Fig 8:**
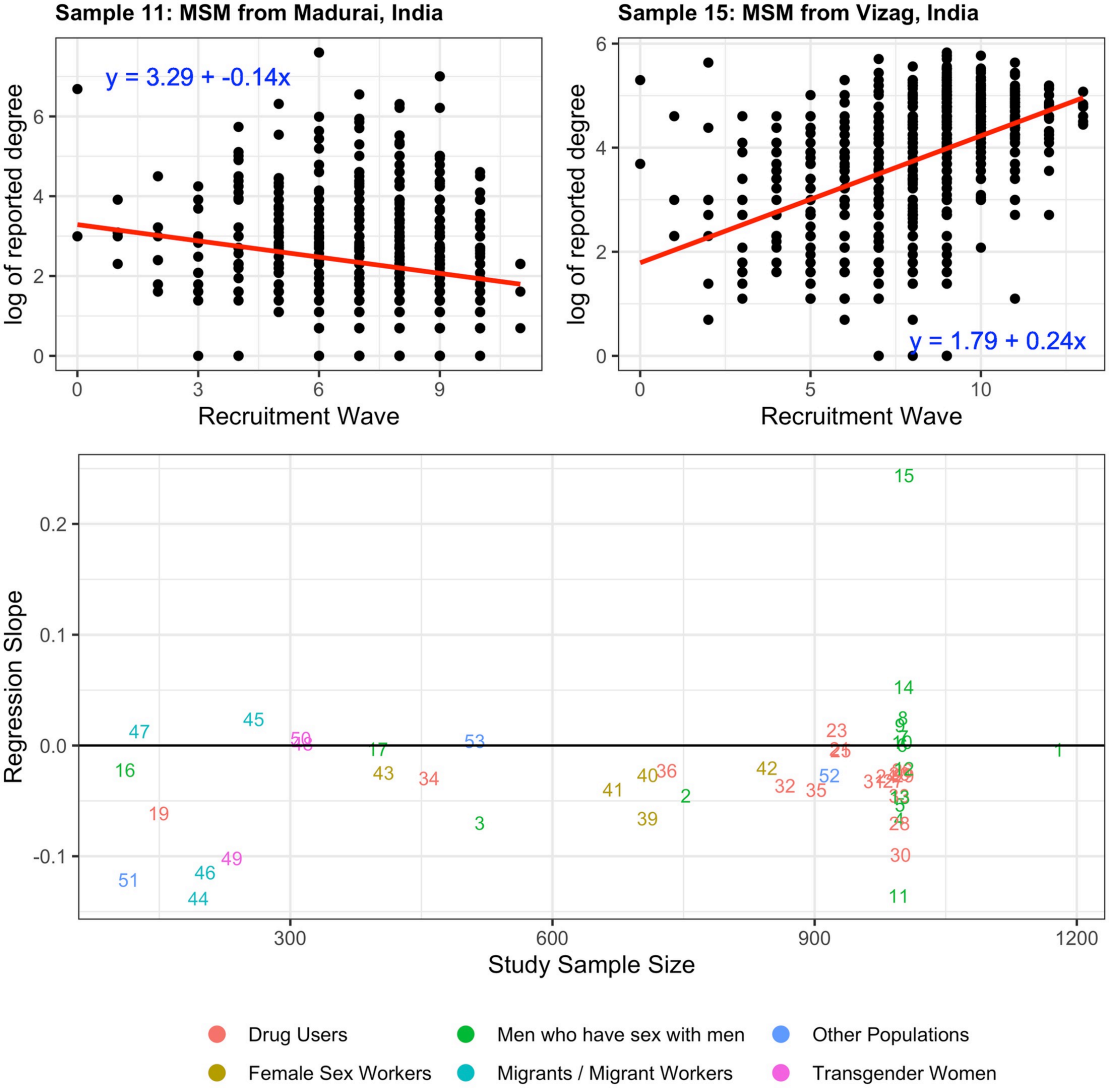
Change in degree over time. Change in logarithm of reported network degree, plotted as a function of sample size. Negative values indicate that the reported degree declined with successive waves. Numbers refer to the sample numbers specified in Table 1.

A reduction in reported degree as study recruitment progress can indicate that a large fraction of the target population has been recruited. Fig 8 illustrates the log of the reported network degrees, as a function of recruitment wave, for the samples experiencing the greatest increase and decrease in reported degree over successive waves. The slopes of the linear regression of the log of degree on wave are shown in the bottom Fig 8, plotted against study sample size, for every study. For most studies, there is little change in reported degree over wave, the study with the largest decline had a rate of change of -0.14 log(degree)/wave, amounting to twenty fewer network connections over ten waves of recruitment.

## 4 Discussion

Details about RDS recruitment chains and reported degrees were collected and summarised for 53 samples, encompassing 36,547 participants representing several target populations in 12 countries. To our knowledge, this is the first study to examine the distribution of RDS-reported degrees across several countries and target populations. Our findings have implications for applied researchers using RDS and for statisticians working to improve estimates arising from these data. Reported degree is a discrete variable, but is best described by a normal distribution on the log-transformed degrees and reports of very high degrees are common. Instead of interpreting individuals with large reported degrees as outliers, the possibility of individuals acting as ‘super nodes’ arises and thoughtful consideration needs to be given before modifying or removing these values. In their work estimating population size from RDS data, Crawford et al. [18] report removing a subject with reported degree of 200 who was considered an outlier. Our results indicate that this is likely too small of a limit for truncation and we suggest caution in modifying or removing data. Sensitivity analysis, in which results with all reported degrees are compared to results with a truncated upper limit on degree may be useful to inform researchers about the effects of highly connected participants on RDS estimates of prevalence.

These findings indicate that reported degree is not a precise measure of actual degree; nearly all degrees greater than ten are reported to the nearest five or ten people. This does not suggest that reported degree is inaccurate, only that it is imprecise. Although reports of degree greater than 1000 may initially seem unlikely, for an individual who has been closely involved with community members over several years 1000 connections is not unreasonable. Given that 14 of the 53 samples reported degrees in excess of 1000 suggests that these large network connections are real. We can not comment further based on the data collected, but future work may focus more on the accuracy of reported degree and its importance in analysing RDS data.

For statisticians developing RDS estimators, the distribution of reported degrees may have implications for estimator accuracy and precision. Statistical tests of the appropriateness of different distributions, such as the Shapiro-Wilk for normal data are not useful for RDS data because of the tendency for reported degree to be reported to the nearest multiple of 5 or 10. For this reason, we compared the likelihood of several reasonable candidate distributions: the Poisson, geometric, negative binomial, discrete q-exponential, Poisson-lognormal, Conway-Maxwell-Poisson, Yule and Waring, as well as the continuous normal distribution on both raw and log-transformed degree. Although a Poisson counting process may seem like a natural mechanism for modelling connectedness, our results indicate that this is the least appropriate distribution for describing degree. Results based on Poisson-simulated degree, such as Fellows’ [10] simulations of the performance of the homophily configuration graph for prevalence estimation, may need to be re-examined in light of what we are now learning about degree distributions in practice.

In our earlier work [14], we showed that weighted regression methods are not suitable for RDS data when the degree distribution is highly skewed, and so we reiterate the need for caution when applying weighted regression methods to RDS data. Extremely skewed degree data results in people with very few reported connections receiving high Volz-Heckathorn weights. These individuals then act as leverage points, and can either nullify true relationships or introduce relationships where none exist. While alternative weighting strategies could be employed, our words of warning stem from the common use of the RDS-II (Volz-Heckathorn) weights in regression analyses of RDS samples.

Long recruitment chains are desirable to minimise the impact of the initial seeds on the final sample. Simulation studies have shown that even with heavily biased seeds, only four or five waves are necessary to ensure unbiased prevalence estimates [1]. Papers often report the length of the longest recruitment chain, but we have not found information regarding wave distribution in the literature. In the samples observed here, all studies that reported maximum chain length of at least eight waves had five or more waves for the median recruit (Fig 5). Studies offering only two coupons per participant achieved the longest chains, while those offering five or more were the least likely to have a median chain length of at least five waves. We encourage authors to report the wave of the median participant, ordered by recruitment timing, as a measure of the potential dependency of the sample on the initial seeds.

There is some evidence that the definition of network ties dampens the degree reports but the shape of the distribution, and the overall spread appear less affected (Fig 7). The implications of tie definition on RDS estimators is beyond the scope of the current study, but requires further investigation.

We found no evidence that the pool of available recruits was depleted by the recruitment process for the samples investigated here. Although Fig 8 indicates that the reported degree often decreases as recruitment progresses (negative values indicate an inverse relationship between degree and wave), the magnitude of the decline is minimal, and there were a number of studies with sample size near 1,000 where the reported degree actually increased with successive waves.

A limitation inherent in any survey is response bias and this survey is no exception. We can not evaluate the distribution of the reported degree for the samples that were not shared. However, we feel that the breadth of sample types (MSM, FSW, PWID, people of colour, migrant workers) and the geographical coverage of the samples (North America, Europe, Africa, Asia and Central America), coupled with the fact that all samples were best described by a normal distribution on log-transformed degree is sufficient to be confident that our results are generalizable. Future work will evaluate the impact of extremely skewed degree on the accuracy of RDS prevalence estimators.

A valid RDS study will achieve a representative sample of the target population, and accurately estimate the disease burden in that community. For researchers employing RDS methods we have two key findings: 1) fewer coupons per participant may be useful in achieving longer recruitment chains and 2) reports of very high network degrees are relatively common, and what constitutes an outlier is unclear. For researchers investigating RDS estimator performance, we recommend using log normal distributions for reported degrees, and recognising that degree is likely to be imprecisely reported by participants. Methodological work on appropriate methods for RDS data will be most informative if validation is undertaken using data that reflects what is observed in practice.

## Supporting information

S1 Data (XLSX)
